# Risk factors for deep venous thrombosis in spontaneous intracerebral hemorrhage and their long-term prognostic implications

**DOI:** 10.1515/med-2025-1341

**Published:** 2026-02-11

**Authors:** Xianju Liang, Chuyue Wu, Yu Huang, Jing Wang, Lei He, Wei Feng

**Affiliations:** Department of Neurology, Chongqing University Three Gorges Hospital, Chongqing, China; School of Medicine, Chongqing University, Chongqing, China; Chongqing Municipality Clinical Research Center for Geriatric Diseases, Chongqing University Three Gorges Hospital, Chongqing, China

**Keywords:** spontaneous intracerebral hemorrhage, deep venous thrombosis, risk factors, functional prognosis

## Abstract

**Objectives:**

This study sought to identify the risk factors associated with venous thrombosis for spontaneous intracerebral hemorrhage (ICH) in a long term.

**Methods:**

A retrospective analysis was performed on the ICH patients between January 2021 and December 2022.

**Results:**

A total of 737 patients were examined with 86 patients (11.7 %) of venous thrombosis. The risk factors linked to venous thrombosis were the length of hospital stay (p<0.001), admission NIH Stroke Scale (p=0.008), and D-dimer levels (p=0.041). No significant differences in mortality rates existed at any timepoint in this study between patients with and without venous thrombosis after correction analysis (p>0.05). However, patients who developed a venous thrombosis exhibited a higher rate of poor outcomes compared to patients who did not develop a venous thrombosis (p<0.05) at the time of hospital discharge (90.7 % vs. 59.6 %), 90 days post-onset (73.2 % vs. 41.6 %), and 1-year post-onset (73.2 % vs. 41.0 %).

**Conclusions:**

The identified risk factors for venous thrombosis among ICH patients include extended hospitalization, elevated NIH Stroke Scale score and D-dimer level. The occurrence of a venous thrombosis correlated with inferior functional outcomes at the time of hospital discharge, at 90 days and 1 year after onset.

## Introduction

Stroke was responsible for 6.6 million deaths and 143 million disabilities in 2019, making it the second most common cause of mortality and morbidity globally [1]. Over the past 3 decades there has been a significant increase in the incidence and fatality rate of strokes worldwide, with a 70 % rise in stroke cases and a 43 % increase in stroke-related deaths [2].

Patients with intracerebral hemorrhage (ICH) often become immobile, which increases the risk of venous stasis and subsequent thrombotic events [3]. Immobility hinders blood flow, promotes clot formation, and elevates the incidence of venous thrombosis (VTE). Patients with ICH have a 2–4-fold higher risk of VTE compared to patients with ischemic stroke. VTE occurs in 2 %–9 % of hospital admissions, and if left untreated, can result in a mortality rate as high as 26 % [4].

The heightened susceptibility to VTE in patients with ICH arises from multiple contributing factors [5]. These factors include male gender and Black/African American ethnicity, which are linked to an increased risk. Clinical conditions, such as infection, a high National Institute of Health stroke scale score (≥12), and an elevated D-dimer level at the time of hospital admission, also contribute to this risk [6]. Additionally, medical interventions and histories, such as the utilization of external ventricular drainage, a history of venous thromboembolism, and intubation, further increase the likelihood of VTE. Furthermore, intraventricular hemorrhage has been established as a significant risk factor for VTE [7].

VTE significantly influences the clinical outcomes and prognosis of patients with ICH. Recent research has focused on identifying the risk factors associated with VTE in this context [3], [4], [5], [6], [7]. Patients with ICH have a significantly higher risk of VTE, primarily due to restricted mobility, coagulation disorders, and inflammatory responses [3], 4]. VTE not only increases the risk of deep vein thrombosis (DVT) and pulmonary embolism (PE) but can also exacerbate neurological damage and mortality [5], [6], [7]. Early preventive measures, such as mechanical compression or anticoagulation therapy, are crucial; Moreover, it is essential to balance the risks of bleeding and thrombosis. Studying the mechanisms and intervention strategies for VTE is crucial for improving the prognosis of ICH patients. The current study aimed to determine these risk factors during hospitalization of patients with spontaneous ICH and evaluate the long-term prognostic implications of VTE. By elucidating these factors, management strategies and clinical outcomes for patients with ICH complications will be improved.

## Methods

### Study design

This retrospective, single-center cohort study was conducted at the Department of Neurology, Chongqing University Three Gorges Hospital, with research protocols approved by the Clinical Trial Ethics Committee of the same institution (No. 20230242). Informed consent was obtained from all participants or their legal representatives. This study followed the Declaration of Helsinki recommendations and registered on the National Medical Research Registration and Archival Information System: https://www.medicalresearch.org.cn; Unique identifier: MR-50-24-001923.

### Participant information

Patients admitted to the Department of Neurology at Chongqing University Three Gorges Hospital between January 2021 and December 2022 were included if they satisfied the diagnostic criteria for ICH outlined in the 2023 *Chinese Guidelines for the Diagnosis and Treatment of ICH*. All patients underwent diagnostic imaging tests, including computed tomography angiography, computed tomography venography, or magnetic resonance imaging. The inclusion criteria included the following: 1) > 18 years of age and not pregnant; and 2) documented deep venous thrombosis (DVT) evaluation parameters at the time of hospital admission and during hospitalization, including specialized physical examinations, biochemical markers, imaging studies, and similar evaluations. The exclusion criteria included the following: 1) secondary hemorrhagic causes, such as tumors, trauma, and cerebral infarction with hemorrhage transformation; 2) a craniotomy during hospitalization; 3) unidentified origin of hemorrhage or presence of multiple simultaneous ICH sites; 4) exclusive ventricular hemorrhage or subarachnoid hemorrhage; 5) DVT before admission; 6) rapid disease progression of *ICH* resulting in death within 24 h; and 7) discharge >1 d after admission.

### Clinical data

All clinical data were extracted from the medical records system: demographic details (gender and age), treatment modalities (limited to medical intervention and minimally invasive surgery [MIS] + medical treatment), time elapsed (hours) from the initial CT scan to symptom onset (defined as the time from symptom onset or sudden exacerbation-to-the start of the first CT examination in the emergency room, outpatient setting, or inpatient ward), length of hospital stay (days), medical history (hypertension, diabetes, cancer, cigarette smoking, and alcohol consumption), admission data (height, weight, and vital signs), classification based on structural vascular lesions, medications, amyloid angiopathy, systemic diseases, hypertension, and undetermined causes (SMASH-U) criteria, and admission evaluation scores (pre-stroke modified Rankin score [mRS], post-stroke mRS, Glasgow Coma scale [GCS], NIH Stroke Scale [NIHSS], activities of daily living [ADL], International Classification of Diseases-antiphospholipid syndrome [ICD-APS], Nutritional Risk Screening 2002 [NRS2002], water swallow test, Padua score, Acute Physiology and Chronic Health Evaluation [APACHE] II score, and Modified Early Warning Score [MEWS]).

### Imaging evaluation

Hemorrhage location was classified as follows: lobar; deep; cerebellar; brainstem [8]; and mixed hematoma (cases with ≥2 of the above categories). Furthermore, the assessment included hemorrhage into the ventricle [9] and subarachnoid space [10]. Hematoma volume was computed in mL using an ellipsoid formula (A × B × C/2) [9], 10]. All imaging studies were evaluated by two neuroradiologic specialists, who were not informed of the clinical details. In cases of disagreement, A third expert was asked to arbitrate and finalize the classification in cases in which the two neuroradiologists disagreed.

### DVT evaluation

A DVT diagnosis required clinician assessment of patient symptoms (swelling, edema, redness, tenderness, and the presence of superficial lateral veins), biomarkers (D-dimer), and imaging tests (ultrasound, Doppler ultrasound, and venography) [11]. Our evaluation specifically examined incidents of DVT upon hospital admission based on routine screening and during hospitalization with the analysis concentrating solely on cases that emerged post-admission. A repeated imaging during hospitalization was proceeded to detect DVT again. All patients who were confirmed of DVT received DVT treatment, such as a pressurized device or anticoagulant, within 24 h of hospital admission with or without undergoing MIS.

### Patient follow-up

Telephone follow-up evaluations were performed to determine patient mortality during the follow-up period. The mRS score for patients with ICH was evaluated at 90 days or 1 year after onset. An mRS score <3 combined with patient healed status was defined as a good prognosis, while an mRS score ≥3 with poor patient status was considered a poor prognosis, including six points, which represented death.

### Statistical analysis

Statistical analyses were performed using SPSS software (version 22). Categorical variables are presented as frequencies and percentages and evaluated using the chi-squared test. Continuous variables are described as the mean ± standard deviation or median with interquartile range (IQR). Group comparisons were performed using the Mann‒Whitney U test or *t*-test.

The cases were first classified into DVT and non-DVT groups to compare the distribution of baseline data. For all the original variables included in the study, independent sample *t*-tests/analysis of variance were used for continuous variables (such as age, BMI, etc.), and chi-square tests were used for categorical variables (such as gender, etiological classification, etc.). Set the test level α=0.10 and retain the variables with p<0.10. Variables with p<0.10 were included in the univariate analysis. Then, the stepwise backward selection method was adopted for the screening of the retained variables with p<0.10. Minimize the value of Akaike information criterion as the final model criterion. Subsequently, multivariate regression analysis was performed with DVT as a binary outcome indicator to determine independent influencing factors. Whether DVT was a binary outcome indicator (yes=1, no=0) was used to perform multivariate regression analysis to determine independent influencing factors. The variance inflation factor (VIF) of the final models was all<5, eliminating the problem of multicollinearity. In the model, “no complications occurred” was taken as the reference group, the core variables after screening were included as independent variables, and the Hosmer-Lemeshow test was used to verify the goodness of fit of the model (test results) χ^2^=3.58, p=0.018, suggesting a good model fit. At the same time, the odds ratio (OR) and 95 % confidence interval (95 % CI) of each variable were calculated to reflect the intensity of the influence of independent variables on the outcome. The differences in prognostic information between the DVT and non-DVT groups were then determined, followed by a correction analysis. A p<0.05 was defined as statistical significance.

## Ethics approval and informed consent

This retrospective, single-center cohort study was conducted at the Department of Neurology, Chongqing University Three Gorges Hospital, with research protocols approved by the Clinical Trial Ethics Committee of the same institution (No. 20230242). Written informed consent was obtained from all participants or their legal representatives following the Declaration of Helsinki (National Medical Research Registration and Archival Information System: https://www.medicalresearch.org.cn; Unique identifier: MR-50-24-001923). No vulnerable patients were included in the study.

## Results

### Demographic data

**Figure 1:**
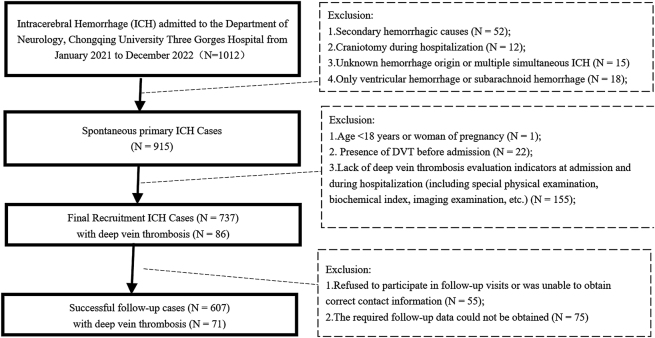
Flow diagram of eligibility of ICH cases.

The flow diagram in Figure 1 outlines the eligibility criteria for patients with ICH. A total of 737 patients diagnosed with ICH were included in this investigation. DVTs occurred in 86 patients (11.7 %), there were 70 in-hospital deaths (9.5 %), and follow-up evaluations were completed in 607 patients (82.4 %). The demographic composition of the cohort consisted of 453 males (61.5 %) and 284 females (38.5 %) with an average age of 67 years (IQR, 16 y; age range, 57–73 y). Treatment included minimally invasive procedures in 226 patients (30.7 %) and pharmacologic intervention in 511 patients (69.3 %). Gastrointestinal bleeding was documented in 102 patients (13.8 %) during the hospitalization period.

The median time from symptom onset-to-hospital admission was 9.11 h (IQR, 7 y). The average hospital stay was 14.19 ± 9.687 d. The mean bleeding volume was 21.54 ± 22.07 mL. The SMASH-U etiologic typology was as follows: vascular structural lesions, 3.0 %; drug-induced lesions, 1.6 %; amyloid angiopathy, 3.0 %; systemic disease, 1.6 %; hypertension, 81.1 %; and unknown causes, 9.6 %. The distribution of hemorrhage sites was as follows: lobe, 11.7 %; deep, 67.4 %; cerebellum, 5.8 %; brainstem, 4.3 %; and multiple sites, 10.7 %. Hemorrhage extended into the ventricles and subarachnoid space in 31.8 % and 7.7 % of patients, respectively.

The medical records indicated the following historical disease data: hypertension, 492 patients (66.8 %); diabetes mellitus, 61 patients (8.3 %); coronary heart disease, 68 patients (9.2 %); atrial fibrillation, 23 patients (3.1 %); cerebral infarction, 45 patients (6.1 %); a history of anticoagulant medication, 31 patients (4.2 %); antiplatelet medication, 33 patients (4.5 %); a history of brain or spinal surgery, 32 patients (4.4 %); cancer, 23 patients (3.1 %), and past gastrointestinal disorders, 73 patients (9.9 %). Cigarette smoking and alcohol consumption were documented in 242 (32.8 %) and 229 patients (31.1 %), respectively.

The patients characteristics were as follows height, 160 cm (IQR, 12 cm); abdominal circumference, 84 cm (IQR, 16 cm); weight, 60 kg (IQR, 16 kg); temperature, 36.6 °C (IQR, 0.3 °C); respiration rate, 19 breaths/min (IQR, 4 breaths/min); heart rate, 78 beats/min (IQR, 21 beats/min); oxygen saturation, 99 % (IQR, 2 %); random glucose level at the time of hospital admission, 6.90 mmol/L (IQR, 1.70 mmol/L); systolic blood pressure, 167 mmHg (IQR, 34 mmHg); and diastolic blood pressure, 92 mmHg (IQR, 22 mmHg).

The median pre- and post-stroke mRS scores at the time of hospital admission were 0 and 4 (IQR, 0 and 1), respectively. The hospital admission GCS score was 13 (IQR, 7) and the NIHSS score was 12 (IQR, 12). The median hospital discharge mRS score was 3 (IQR, 2), indicating a poor prognosis in 466 patients (63.2 %). The hospital discharge GCS score was 14 (IQR, 5) and the NIHSS score was 8 (IQR, 13). Additional scores included the following: ADL, 20 points (IQR, 39); ICD-APS, seven points (IQR, 5); NRS2002, three points (IQR, 1), water swallow test grade, 3 (IQR, 4); Padua score, three points (IQR, 1); APACHE II score, 10 points (IQR, 9), and MEWS, two points (IQR, 3).

There were 30 (4.9 %) and 44 deaths (7.2 %) at the 90-d and 1-y follow-up evaluations, respectively. The median mRS score at the 90-d follow-up evaluation was two points (IQR, 3), indicating an unfavorable prognosis for 275 patients (45.3 %). The median mRS score at the 1-y follow-up evaluation was two points (IQR, 2), indicating a poor prognosis for 266 patients (43.8 %).

**Figure 2:**
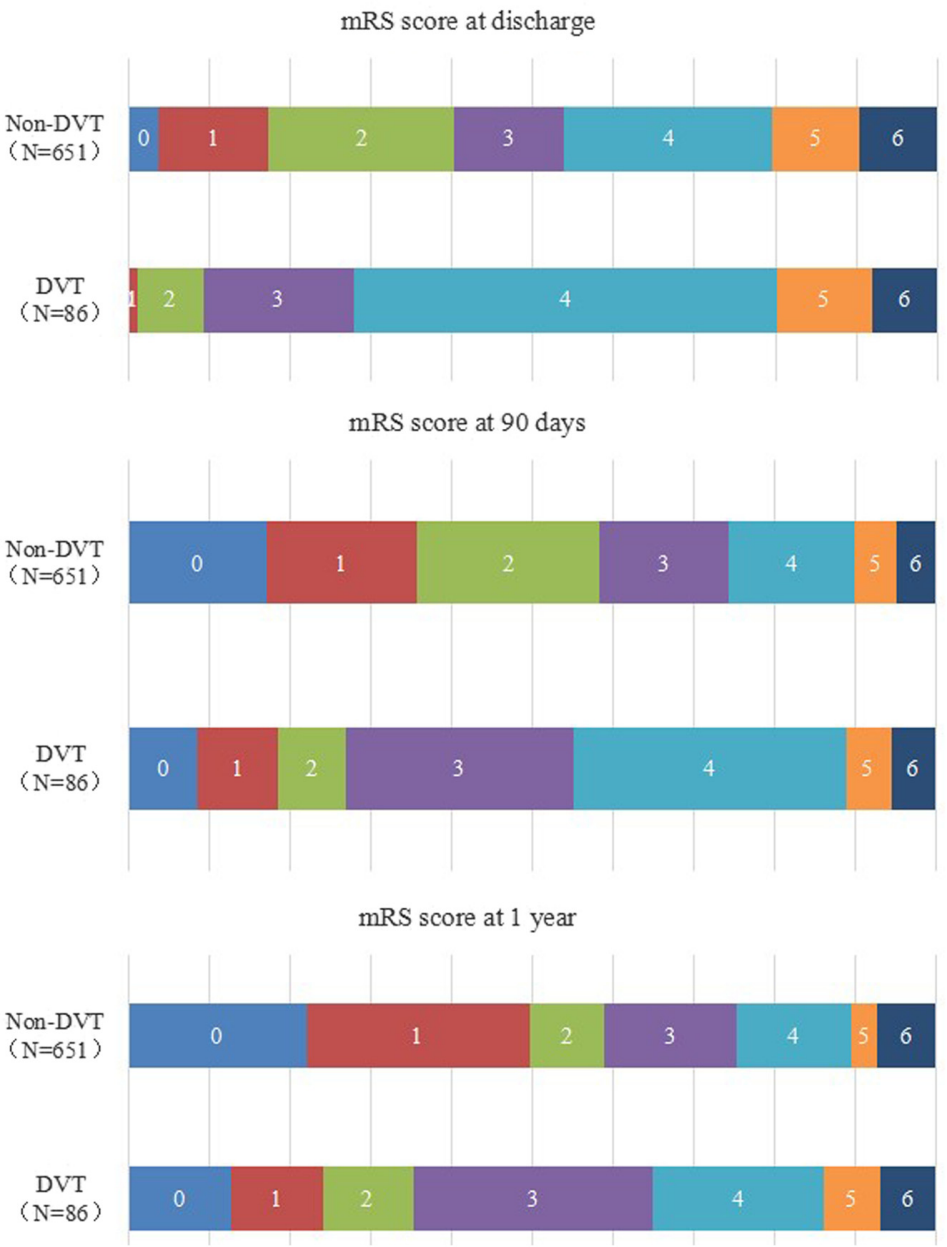
The mRS scores between the DVT and non-DVT groups at different periods for ICH cases.

Figure 2 shows the bar charts of 0–6 segments of mRS scores at discharge, 90 days and 1 year after discharge between non-DVT and DVT groups.

### Differences in characteristics between the non-DVT and DVT groups

Significant differences existed in patients with a DVT compared to patients without a DVT with respect to age (p=0.025), treatment modality (p=0.002), length of hospital stay (p<0.001), in-hospital gastrointestinal bleeding (p=0.043), history of hypertension (p=0.036), history of coronary heart disease (p=0.045), history of atrial fibrillation (p<0.001), SMASH-U classification (p=0.025), mRS after ICH (p<0.001,), GCS (p=0.002), NHISS (p<0.001), ADL (p<0.001), ICD-APS (p<0.001), NRS2002 (p<0.001), water swallow test (p<0.001), Padua score (p=0.002), APACHE II score (p<0.001), MEWS (p=0.036), hematoma volume (p=0.029), platelet count (p=0.036), total bilirubin level (p=0.035), indirect bilirubin level (p=0.046), total cholesterol level (p=0.047), lipoprotein A level (p=0.041), hemoglobin A1C concentration (p=0.032), myoglobin level (p=0.040), D-dimer level (p=0.009), and fibrin degradation product level (p=0.026), as shown in Table 1. Supplemental Table 1 shows the complete data about the differences of the characteristics between the non-DVT and DVT groups.

**Table 1:** Significant Differences in characteristics between non-DVT and DVT group.

Characteristic data	Variables		DVT (n=86) n(%)/Mean ± SD/Median(IQR)	Non-DVT (n=651) n(%)/Mean ± SD/Median(IQR)	p-Value	OR/T index/U index
Demographic						
	Age, years		70(16)	66(17)	0.025^a^	2.246
	Treatment modality	Only medical	47(54.7 %)	464(71.3 %)	0.002^a^	2.059
		MIS+ medical	39(45.3 %)	187(28.7 %)		
	Length of hospitalization, days		17(11)	12(9)	<0.001^a^	7.065
	In-hospital gastrointestinal bleeding	0	68(79.1 %)	567(87.1 %)	0.043^a^	1.787
		1	18(20.9 %)	84(12.9 %)		
Past history						
	Hypertension	0	20(23.3 %)	225(34.6 %)	0.036^a^	1.743
		1	66(76.7 %)	426(65.4 %)		
	Coronary heart disease	0	73(84.9 %)	596(91.6 %)	0.045^a^	1.930
		1	13(15.1 %)	55(8.4 %)		
	Atrial fibrillation	0	78(90.7 %)	636(97.7 %)	<0.001^a^	4.349
		1	8(9.3 %)	15(2.3 %)		
		1	23(26.7 %)	206(31.6 %)		
Admission data	SMASH-U classification	Structural lessons	1(1.2 %)	21(3.2 %)	0.025^a^	2.238
		Medication	3(3.5 %)	9(1.4 %)		
		Cerebral amyloid angiopathy	2(2.3 %)	20(3.1 %)		
		Systemic disease	1(1.2 %)	11(1.7 %)		
		Hypertension	78(90.6 %)	520(79.9 %)		
		Undetermined	1(1.2 %)	70(10.8 %)		
					
	mRS after ICH		5(1)	4(2)	<0.001^a^	5.256
	Glasgow Coma Scale		9(7)	13(7)	0.002^a^	−3.164
	NIH Stroke Scale		16(7)	11(13)	<0.001^a^	4.883
	Activities of daily living		20(20)	25(40)	<0.001^a^	−3.582
	International classification of diseases-antiphospholipid syndrome		9(4)	6(5)	<0.001^a^	5.165
	NRS2002		3(1)	3(1)	<0.001^a^	3.998
	Water swallow test		5(2)	3(4)	<0.001^a^	4.675
	Padua		4(2)	3(1)	0.002^a^	3.086
	Acute Physiology and Chronic health Evaluation-II		14(8)	9(9)	<0.001^a^	4.620
	Modified early warning score		2(2)	2(3)	0.036^a^	2.091
CT imaging data						
	Hematoma volume, mL		20.00(18.29)	15.00(21.80)	0.029^a^	2.177
Serum biochemical evaluations						
Platelet, ^a^10^b^9/L			201(75)	184(78)	0.036^a^	2.092
Total bilirubin, umol/L			8.40(8.20)	10.10(6.90)	0.035^a^	−2.112
Indirect bilirubin, umol/L			4.90(5.50)	6.00(4.60)	0.046^a^	−1.995
Total cholesterol, mmol/L			4.09(1.23)	4.42(1.25)	0.047^a^	−1.984
Lipoprotein A, mmol/L			32.10(59.19)	21.60(38.30)	0.041^a^	2.040
Hemoglobin A1C, %			5.77(0.58)	5.60(0.42)	0.032^a^	2.145
Myoglobin, ng/m			94.29(266.82)	58.65(108.21)	0.040^a^	2.049
D-dimer, mg/L			0.73(1.25)	0.49(0.76)	0.009^a^	2.610
Fibrin degradation products, mg/L			1.65(2.58)	1.32(1.92)	0.026^a^	2.222

^a^p<0.05. ^b^The time from admission to onset was determined as the duration between the precise moment when symptoms first appeared or when the patient was last observed to be in a normal state upon arrival at the emergency, outpatient, or inpatient setting. ICH, intracerebral hemorrhage; DVT, deep venous thrombosis; mRS, modified Rankin score.

Independent risk factors associated with the presence of a DVT based on multivariate analysis included the length of hospital stay (adjusted [Adj.] p<0.001, OR=1.115, 95 % confidence interval [CI]=1.053–1.180), admission NIHSS (Adj. p=0.008, OR=1.059, 95 % CI=1.029–1.089), and D-dimer level (Adj. p=0.041, OR=1.031, 95 % CI=1.000–1.063), as shown in Table 2. The parametric indicators for evaluating the regression model integrating independent factors were as follows:Sensitivity=0.862, Specificity=0.693, Youden’s index=0.555, Positive predictive value=10.81 %, Negative predictive value=91.32 %. The AUC of ROC curve was 0.826. We subsequently re-analyzed the data using the competitive risk model (Fine-Gray model), taking “in-hospital death” as the competitive event and “in-hospital occurrence of VTE” as the outcome event, to re-verify the correlation of risk factors. The results in Table 3 showed that, after including the competitive risk of “death”, the association strength (OR/HR value) and statistical significance (95 % CI that did not include 1) in the core risk factors (length of hospital stay, NIHSS score at admission, D-dimer level) with VTE did not show substantial changes. The results show that despite the existence of “death competitive risk”, the core risk factors identified in this study still have stability and reliability.

**Table 2:** The multivariate analysis between non-DVT and DVT groups.

Risk factors	B	S.E.	Wald	df	Adj.P	OR	95 % CI	Adj.OR	Adj.95 % CI
Length of hospitalization, days	0.109	0.029	14.092	1	<0.001	1.115	1.053, 1.180	1.252	1.181–1.426
Admission NIH Stroke Scale	0.057	0.014	15.709	1	0.008	1.059	1.029, 1.089	1.082	1.021–1.174
D-dimer, mg/L	0.030	0.016	3.809	1	0.041	1.031	1.000, 1.063	1.053	1.001–1.283

Adj. is the reanalysis of the data using the competing risk model (fine-gray model), with “in-hospital death” as the competing event and “in-hospital VTE” as the outcome event to revalidate the association of risk factors.

**Table 3:** The results of competitive risk model (fine-gray).

Risk factors	Primitive logistic regression (OR, 95 % CI)	Competitive risk model (HR, 95 % CI)	Result consistency
Length of hospitalization, days	1.115(1.053, 1.180)	1.252(1.181–1.426)	Highly consistent
Admission NIH Stroke Scale	1.059(1.029, 1.089)	1.082(1.021–1.174)	Highly consistent
D-dimer, mg/L	1.031(1.000, 1.063)	1.053(1.001–1.283)	Highly consistent

### Comparison of prognosis in ICH patients between the DVT and non-DVT groups at different times

The DVT group had a comparable mortality rate during hospitalization (7[8.1 %]) with the non-DVT group (63[9.7 %]; Adj. p=0.648 and Adj. OR=0.827). The DVT group had a higher prevalence of poor prognosis at the time of hospital discharge compared to the non-DVT group (78 [90.7 %] vs. 388 [59.6 %]; Adj. p<0.001 and Adj. OR=6.609). Additionally, notable differences were noted in the mRS score (Adj. p<0.001, Adj. T=4.264), NIHSS score (Adj. p<0.001, Adj. T=4.550), and GCS score (Adj. p=0.001, Adj. T=3.434). Further details are summarized in Table 4.

**Table 4:** Comparison of prognosis in ICH cases between DVT and non-DVT groups at different periods.

Discharge n=737		DVT (n=86) n(%)/mean ± SD/Median(IQR)	Non-DVT(n=651) n(%)/Mean ± SD/Median(IQR)	Adj.P^a^	Adj.OR/T value^a^
mRS		4(3);3.56 ± 1.515	2(3);2.49 ± 1.506	<0.001	4.264
NIH Stroke Scale		10(13);12.52 ± 10.514	2(7);5.78 ± 8.401	<0.001	4.550
GCS		13(7);11.12 ± 4.169	15(1);13.68 ± 2.995	0.001	3.434
Death during hospitalization	0	79(91.9 %)	588(90.3 %)	0.648	0.827
	1	7(8.1 %)	63(9.7 %)		
Prognosis	Good^b^	8(9.3 %)	263(40.4 %)	<0.001	6.609
	Poor^b^	78(90.7 %)	388(59.6 %)		
At 90 days n=607		DVT(n=71) n(%)/Mean ± SD/Median(IQR)	Non-DVT(n=536) n(%)/Mean ± SD/Median(IQR)	Adj.P^a^	Adj.OR/T value^a^
mRS		3(3);2.67 ± 1.733	2(2);1.84 ± 1.424	<0.001	4.019
Death	0	67(94.4 %)	510(95.1 %)	1.000	1.171
	1	4(5.6 %)	26(4.9 %)		
Prognosis	Good^b^	19(26.8 %)	313(58.4 %)	<0.001	3.841
	Poor^b^	52(73.2 %)	223(41.6 %)		
At 1 year n=607		DVT(n=86) n(%)/Mean ± SD/Median(IQR)	Non-DVT (n=651) n(%)/mean ± SD/median (IQR)	Adj.P^a^	Adj.OR/T value^a^
mRS		3(3);2.56 ± 1.865	1(3);1.51 ± 1.523	0.001	3.362
Death	0	66(93.0 %)	497(92.7 %)	0.943	0.965
	1	5(7.0 %)	39(7.3 %)		
Prognosis	Good^b^	25(35.2 %)	316(59.0 %)	<0.001	2.643
	Poor^b^	46(64.8 %)	220(41.0 %)		

^a^p<0.05, statistically significant difference compared with the control group. ^b^An mRS score of less than 3 was defined as a good prognosis, and the rest had a poor prognosis (death (6 points) was also included). ICH, intracerebral hemorrhage; DVT, deep venous thrombosis; mRS, modified Rankin score; GCS, Glasgow Coma Scale.

The DVT group had a comparable mortality rate 90 d after onset (4 of 5.6 %) with the non-DVT group (26 of 4.9 %; Adj. p=1.000 and Adj. OR=1.171). The DVT group had a greater prevalence of poor prognosis 90 d after onset (73.2 % vs. 41.6 %, Adj. p<0.001, Adj. OR=3.841) compared to the non-DVT group. Additionally, significant differences were noted in the mRS scores between the two groups (Adj. p<0.001, Adj. T=4.019).

The DVT group had a comparable mortality rate 1 y after onset (5 of 7.0 %) with the non-DVT group (39 of 7.3 %; Adj. p=0.943 and Adj. OR=0.965). Conversely, the DVT group had a higher incidence of poor prognosis (64.8 % vs. 41.0 %; Adj. p<0.001 and Adj. OR=2.643). Moreover, significant differences were detected in mRS scores (Adj. p=0.001 and Adj. T=3.362).

## Discussion

The identified risk factors for DVT among ICH patients include extended hospitalization, elevated NIHSS score and D-dimer level. While DVT did not influence the mortality rate, the occurrence of DVT correlated with inferior functional outcomes at the time of hospital discharge and at 90 days and 1 year after onset.

DVT is a frequent complication of stroke. A meta-analysis performed by Zeng et al. [12] revealed that the incidence of concurrent venous thromboembolic disease in patients with acute ICH in Asian populations was approximately 8.5 %. In the current study there was a notably higher prevalence of DVT (11.7 %) than that previously documented by Zeng et al. [12]. This disparity can be attributed to three primary factors: first, our study exclusively included patients who underwent DVT assessments, potentially increasing the observed frequency; second, the diagnosis of DVT in the current retrospective study relied on color doppler ultrasound, which may introduce variability due to differences in the proficiency of the sonographers performing the examinations; third, Obesity and advanced age (especially beyond 70 years old), are associated with an increased risk of DVT in patients with ICH [13], [14], [15]. The patients in the current study also had an average old age, which may contribute to a high DVT rate.

Previous investigations underscored DVT as a significant prognostic risk factor in acute ICH patients at the time of hospital discharge and up to 3 months later [16], 17]. The current study showed that patients with DVT had significantly higher rates of adverse outcomes at the time of hospital discharge, 90 d post-onset than the patients without a DVT, which are consistent with previous studies [16], 17]. Moreover, the current study found that patients with DVT had significantly higher rates of adverse outcomes at 1 y post-onset compared to patients without a DVT. This finding highlights the long-term effects of DVT, and underscores the critical importance of prioritizing DVT prevention and early intervention in these patients to improve both long-term prognoses.

The current study pinpointed NIHSS score at the time of hospital admission as an independent factor affecting DVT, which is consistent with previous studies [12], 18]. Moreover, the NIHSS score has indicated a link between neurologic deficits and DVT [19], suggesting that neurologic impairments may contribute to the risk of DVT in patients with ICH. Most international guidelines (such as AHA/ASA, ESO) recommend that patients within 4.5 h of onset of stoke and without contraindications along with the NIHSS score of ≤25 points, receive intravenous thrombolytic therapy [12], 18]. It is recommended to identify the deterioration trend of neurological function through the dynamic NIHSS score [12], 18] while NIHSS is increased.

Ji et al. [11] established a significant correlation between length of hospital stay and the in-hospital DVT in the lower extremities following ICH. Prolonged hospitalization has been identified as a risk factor for DVT in patients with ICH, likely due to the association between extended hospital stays and deteriorating patient conditions leading to increased complications [20]. Our study also indicated the length of hospital stay is the independent factor for DVT, being consistent with the previous researches [11], 20]. These results emphasize the importance of monitoring perioperative conditions of the ICH patients to predict the occurrence of DVT.

Studies have consistently shown a strong correlation between an elevated D-dimer level and the development of DVT in patients with ICH [21], 22]. Additionally, combining the D-dimer level with the albumin level holds promise in effectively predicting concurrent DVT in patients with ICH [22]. The current study found D-dimer level was an independent factor for DVT, the same as previous studies [21], 22]. The results suggested, D-dimer level is an effective biomarker for detecting DVT in clinical practice.

An elevated c-reactive protein level [23], elevated white blood cell count [24], blood glucose and triglyceride levels [25], 26], uric acid [27], 28], Vitamin D levels<30 ng/mL [29] are reported to be related to the patients with a DVT. Although these factors [23], [24], [25], [26], [27], [28], [29] were not found to be significant factor in the multivariable regression analysis of this study, the attention on these factors should be taken.

New strategies are needed to prevent ICH-related DVT. For patients with complications of DVT, they should stand or walk a short distance within 24 h after the operation as much as possible; For patients who are unable to move independently (such as hemiplegia due to stroke), the lower limb flexion and extension movements should be assisted by nursing staff [30], 31]. Meanwhile, an active plus stratified medication regimen should be adopted‌. For ‌ patients at medium and low risk ‌, a preventive dose of low-molecular-weight heparin (such as enoxaparin 40 mg/day) was used; For the very high-risk patients, a combined treatment of anticoagulant drugs and mechanical prophylaxis can be adopted; Mechanical prophylaxis is preferred for those at high risk of bleeding (e.g. within 24 h after surgery), followed by drug prophylaxis and early activity prophylaxis when the risk of bleeding is reduced [30], 31].

The advantage of our research lies in the relatively large sample size cohort (737 cases) with the complete clinical data and the long-term follow up data on the prognosis in this study. Standardized and orderly VTE and various clinical evaluations, which provides strong feasibility of this study. The risk factors related to VTE in this study involve multiple aspects, which have a wide coverage and are highly representative of the population.

However, this study had several limitations. First, single-center retrospective cohort studies are susceptible to biases in patient selection and data collection. Second, this was a retrospective study, and the diagnosis of a DVT relied on assessments by attending physicians at the time of hospital admission, potentially introducing variability due to multiple assessors. Third, the study period coincided with the COVID-19 pandemic, which may potentially influences the study outcomes. Fourth, only one ultrasound assessment for DVT was performed during hospitalization, lacking continuous monitoring on DVT until discharge. The data on thrombus pumps or anticoagulant therapy for DVT prophylaxis and the dynamic monitoring of D-dimer levels were not captured. Fifth, in terms of DVT risk, the subgroup analysis for patients who received only medical treatment vs. those who underwent brain surgery for ICH was not performed due to the lack of data based on the retrospective study. Subsequent research should explore dynamic changes in D-dimer levels and the use of thrombus pumps or anticoagulant therapy to elucidate further factors influencing DVT and the implications on prognosis of patients with acute ICH. In order to further strengthen the robustness of the results, other methods can be considered to address the baseline imbalance problem in further studies, such as propensity score matching or reverse treatment probability weighting. Verification through different statistical methods will ensure the reliability of the conclusions. The time event analysis, such as Kaplan-Meier curves and Cox proportional hazard regression, can be considered in further researches to better capture the timing of DVT occurrence. This will help to better understand the risk over time. Finally, for the model aspect, the original model did not directly incorporate “death” as a competitive risk factor, which may have a minor impact on the VTE risk estimation for a small number of overlapping cases. The interaction between “cause of death and VTE risk” (e.g., whether patients who died from non-cerebral hemorrhage-related causes such as infection or heart failure have different VTE risks compared to those who progressed to death from cerebral hemorrhage) requires further analysis, which could serve as a key focus for subsequent research.

## Conclusions

A DVT is a common complication following ICH that significantly impacts patient’s prognosis at the time of hospital discharge and at 90 days and 1 year after onset. The identified risk factors for DVT among ICH patients include extended hospitalization, elevated NIHSS score and D-dimer level. Future research should prioritize enhancing preventive strategies for elderly patients with high admission NIHSS scores, extended hospital stays, and elevated D-dimer levels. Timely interventions are essential for reducing complications and promoting patient recovery.

## Supplementary Material

Supplementary Material
