# Supplementary material for: Risk factors for deep venous thrombosis in spontaneous intracerebral hemorrhage and their long-term prognostic implications
Source: Open Med (Wars). 2026 Feb 11;21(1):20251341. doi: 10.1515/med-2025-1341 (PMC12917554; doi:10.1515/med-2025-1341)
Supplement: Supplementary file 1 — Supplementary Material [file j_med-2025-1341_suppl_001.docx]

**Supplemental Table 1．Differences in the characteristics between non-DVT and DVT groups**

| Characteristic data | | Variables | | | | | DVT（N=86）  N(%)/Mean±SD/Median(IQR) | Non-DVT（N=651）  N(%)/Mean±SD/Median(IQR) | P | OR/ T index/ U index |
| --- | --- | --- | --- | --- | --- | --- | --- | --- | --- | --- |
| Demographic | |  | | | | |  |  |  |  |
|  | | Age, years | | |  | | 70(16) | 66(17) | 0.025* | 2.246 |
|  | | Gender | | | Male | | 46(53.5%) | 407(62.5%) | 0.106 | 1.450 |
|  | |  |  |  | Female | | 40(46.5%) | 244(37.5%) |  |  |
|  | | Treatment modality | | | Only Medical | | 47(54.7%) | 464(71.3%) | 0.002* | 2.059 |
|  | |  |  |  | MIS+ Medical | | 39(45.3%) | 187(28.7%) |  |  |
|  | Time from admission to onset^^^, hours | | | | | | 4(7) | 5(7) | 0.061 | -1.877 |
|  | | Length of hospitalization, days | | | | | 17(11) | 12(9) | <0.001* | 7.065 |
|  | In-hospital gastrointestinal bleeding | | | | | 0 | 68(79.1%) | 567(87.1%) | 0.043* | 1.787 |
|  |  |  |  |  |  | 1 | 18(20.9%) | 84(12.9%) |  |  |
| Past History | |  | | | |  |  |  |  |  |
|  | | Hypertension | | | | 0 | 20(23.3%) | 225(34.6%) | 0.036* | 1.743 |
|  | |  |  |  |  | 1 | 66(76.7%) | 426(65.4%) |  |  |
|  | | Diabetes | | | | 0 | 76(88.4%) | 600(92.2%) | 0.230 | 1.548 |
|  | |  |  |  |  | 1 | 10(11.6%) | 51(7.8%) |  |  |
|  | | Coronary Heart Disease | | | | 0 | 73(84.9%) | 596(91.6%) | 0.045* | 1.930 |
|  | |  |  |  |  | 1 | 13(15.1%) | 55(8.4%) |  |  |
|  | | Atrial Fibrillation | | | | 0 | 78(90.7%) | 636(97.7%) | <0.001* | 4.349 |
|  | |  |  |  |  | 1 | 8(9.3%) | 15(2.3%) |  |  |
|  | | Ischemic Stroke | | | | 0 | 77(89.5%) | 615(94.5%) | 0.072 | 1.997 |
|  | |  |  |  |  | 1 | 9(10.5%) | 36(5.5%) |  |  |
|  | | Anticoagulant Drug Use | | | | 0 | 80(93.0%) | 626(96.2%) | 0.173 | 1.878 |
|  | |  |  |  |  | 1 | 6(7.0%) | 25(3.8%) |  |  |
|  | | Anti-plate Drug Use | | | | 0 | 81(94.2%) | 623(95.7%) | 0.524 | 1.373 |
|  | |  |  |  |  | 1 | 5(5.8%) | 28(4.3%) |  |  |
|  | | Brain and spine surgery | | | | 0 | 82(95.3%) | 623(95.7%) | 0.904 | 1.085 |
|  | |  |  |  |  | 1 | 4(4.7%) | 28(4.3%) |  |  |
|  | | Prior gastrointestinal disease | | | | 0 | 76(88.4%) | 588(90.3%) | 0.602 | 1.228 |
|  | |  |  |  |  | 1 | 10(11.6%) | 63(9.7%) |  |  |
|  | | Cancer | | | | 0 | 78(90.7%) | 636(97.7%) | 0.068 | 4.349 |
|  | |  |  |  |  | 1 | 8(9.3%) | 15(2.3%) |  |  |
|  | | Smoking | | | | 0 | 58(67.4%) | 437(67.1%) | 0.953 | 0.986 |
|  | |  |  |  |  | 1 | 28(32.6%) | 214(32.9%) |  |  |
|  | | Alcohol Consumption | | | | 0 | 63(73.3%) | 445(68.4%) | 0.356 | 0.789 |
|  | |  |  |  |  | 1 | 23(26.7%) | 206(31.6%) |  |  |
| Admission data | | Height, cm | | | | | 160(12) | 160(12) | 0.680 | -0.412 |
|  |  |  | | | | |  |  |  |  |
|  | | Circumference of abdomen, cm | | | | | 84(18) | 84(16) | 0.512 | 0.655 |
|  | | Weight, kg | | | | | 62(17) | 60(16) | 0.656 | 0.446 |
|  | | Temperature, ℃ | | | | | 36.5(0.3) | 36.6(0.3) | 0.295 | -1.048 |
|  | | Breathe, sub / cent | | | | | 19(5) | 19(4) | 0.508 | -0.662 |
|  | | Heart rate, sub / cent | | | | | 81(23) | 78(21) | 0.372 | 0.892 |
|  | | Degree of blood oxygen, % | | | | | 99(2) | 99(2) | 0.622 | -0.493 |
|  | | Serum glucose levels, mmol/L | | | | | 6.60(2.43) | 6.90(2.90) | 0.558 | -0.586 |
|  | | Systolic blood pressure, mmHg | | | | | 167(31) | 166(34) | 0.744 | 0.326 |
|  | | Diastolic blood pressure, mmHg | | | | | 92(25) | 92(22) | 0.482 | -0.702 |
|  | | SMASH-U Classification | | Structural lessons | | | 1(1.2%) | 21(3.2%) | 0.025* | 2.238 |
|  | |  |  | Medication | | | 3(3.5%) | 9(1.4%) |  |  |
|  | |  |  | cerebral amyloid angiopathy | | | 2(2.3%) | 20(3.1%) |  |  |
|  | |  |  | Systemic disease | | | 1(1.2%) | 11(1.7%) |  |  |
|  | |  |  | Hypertension | | | 78(90.6%) | 520(79.9%) |  |  |
|  | |  |  | Undetermined | | | 1(1.2%) | 70(10.8%) |  |  |
| Admission score | | mRS before ICH | | | | | 0(0) | 0(0) | 0.438 | 0.776 |
|  |  |  | | | | |  |  |  |  |
|  | | mRS after ICH | | | | | 5(1) | 4(2) | <0.001* | 5.256 |
|  | | Glasgow Coma Scale | | | | | 9(7) | 13(7) | 0.002 | -3.164 |
|  | | NIH Stroke Scale | | | | | 16(7) | 11(13) | <0.001* | 4.883 |
|  | | Activities of Daily Living | | | | | 20(20) | 25(40) | <0.001* | -3.582 |
|  | | International Classification of Diseases-antiphospholipid syndrome | | | | | 9(4) | 6(5) | <0.001* | 5.165 |
|  | | NRS2002 | | | | | 3(1) | 3(1) | <0.001* | 3.998 |
|  | | Water swallow test | | | | | 5(2) | 3(4) | <0.001* | 4.675 |
|  | | Padua | | | | | 4(2) | 3(1) | 0.002* | 3.086 |
|  | | Acute Physiology and Chronic Health Evaluation-II | | | | | 14(8) | 9(9) | <0.001* | 4.620 |
|  | | Modified Early Warning Score | | | | | 2(2) | 2(3) | 0.036* | 2.091 |
| CT imaging data | |  |  | | | |  |  |  |  |
|  | | Hematoma location | Lobar | | | | 10(11.6%) | 76(11.7%) | 0.990 | 0.737 |
|  |  |  | Deep | | | | 59(68.6%) | 438(67.3%) |  |  |
|  | |  | Cerebellar | | | | 2(2.3%) | 41(6.3%) |  |  |
|  | |  | Brainstem | | | | 3(3.5%) | 29(4.5%) |  |  |
|  | |  | Mixed hematoma | | | | 12(14.0%) | 67(10.3%) |  |  |
|  | | Hematoma volume, ml | | | | | 20.00(18.29) | 15.00(21.80) | 0.029* | 2.177 |
|  | | Ventricular hemorrhage | | | 0 | | 53(61.6%) | 450(69.1%) | 0.160 | 1.394 |
|  | |  |  |  | 1 | | 33(38.4%) | 201(30.9%) |  |  |
|  | | Subarachnoid hemorrhage | | | 0 | | 81(94.2%) | 599(92.0%) | 0.478 | 0.711 |
|  | |  |  |  | 1 | | 5(5.8%) | 52(8.0%) |  |  |
| Serum biochemical evaluations | | | | | | | | | | |
| White Blood Cells, 10^9/L | | | | | | | 9.76(4.71) | 9.01(4.46) | 0.088 | 1.707 |
| Platelet, *10^9/L | | | | | | | 201(75) | 184(78) | 0.036* | 2.092 |
| C-reactive Protein, mg/L | | | | | | | 2.18(3.69) | 1.81(3.87) | 0.429 | 0.790 |
| Serum Glucose, mmol/L | | | | | | | 6.66(3.04) | 6.87(2.70) | 0.597 | -0.529 |
| Glomerular Filtration Rate, mL/min | | | | | | | 87.50(26.10) | 88.10(21.55) | 0.924 | 0.096 |
| Total bilirubin, umol/L | | | | | | | 8.40(8.20) | 10.10(6.90) | 0.035* | -2.112 |
| Direc bilirubin, umol/L | | | | | | | 3.80(3.10) | 4.30(2.50) | 0.062 | -1.864 |
| Indirect bilirubin, umol/L | | | | | | | 4.90(5.50) | 6.00(4.60) | 0.046* | -1.995 |
| Total cholesterol, mmol/L | | | | | | | 4.09(1.23) | 4.42(1.25) | 0.047* | -1.984 |
| Lipoprotein A, mmol/L | | | | | | | 32.10(59.19) | 21.60(38.30) | 0.041* | 2.040 |
| Hemoglobin A1C, % | | | | | | | 5.77(0.58) | 5.60(0.42) | 0.032* | 2.145 |
| Myoglobin, ng/m | | | | | | | 94.29(266.82) | 58.65(108.21) | 0.040* | 2.049 |
| Prothrombin Time, seconds | | | | | | | 10.55(1.18) | 10.60(1.10) | 0.785 | -0.273 |
| International Normalized Ratio | | | | | | | 0.94(0.10) | 0.93(0.09) | 0.748 | 0.322 |
| Prothrombin Time Activity, % | | | | | | | 111.20(23.95) | 113.10(22.00) | 0.885 | -0.145 |
| Activated Partial Thromboplastin Time, seconds | | | | | | | 24.60(2.70) | 24.75(3.00) | 0.448 | -0.758 |
| Fibrinogen, g/L | | | | | | | 3.04(1.08) | 2.79(0.96) | 0.060 | 1.883 |
| Thrombin Time, seconds | | | | | | | 17.65(1.28) | 17.80(1.40) | 0.153 | -1.431 |
| D-Dimer, mg/L | | | | | | | 0.73(1.25) | 0.49(0.76) | 0.009* | 2.610 |
| Fibrin Degradation Products, mg/L | | | | | | | 1.65(2.58) | 1.32(1.92) | 0.026* | 2.222 |
| Antithrombin, % | | | | | | | 93.00(14.30) | 91.75(18.10) | 0.871 | 0.162 |

* P < 0.05

^ The time from admission to onset was determined as the duration between the precise moment when symptoms first appeared or when the patient was last observed to be in a normal state upon arrival at the emergency, outpatient, or inpatient setting.

& A mix hematoma was identified when a hematoma is situated in two or more areas: lobar, deep, cerebellar, and brainstem.

ICH: intracerebral hemorrhage; DVT: deep venous thrombosis; mRS: modified Rankin score;
